# Complications in a Young Adult Attributable to a Retained Pediatric Dynamic Hip Screw

**DOI:** 10.1155/2019/6814375

**Published:** 2019-07-17

**Authors:** Jonathan Bryant, Leroy Butler, Brandon Green, Daniel Krenk

**Affiliations:** ^1^East Tennessee State University, 130 W Ravine Rd., Suite 9C, Kingsport, TN 37660, USA; ^2^OrthoTexas, 5757 Warren Parkway, POB II, Suite 180, Frisco, TX 75034, USA; ^3^Ballad Health, Johnson City Medical Center, 400 N. State of Franklin Road, Johnson City, TN 37604, USA; ^4^Regional Orthopedic Trauma Associates, 875 Larry Neil Way, Kingsport, TN 37660, USA

## Abstract

**Introduction:**

Orthopedic implants are used for many different conditions in the pediatric population. The literature on hardware removal is controversial and vague.

**Case Report:**

We highlight a young adult male who underwent a dynamic hip screw (DHS) due to a motor vehicle accident at 11 years old. He healed the fracture and did well for years. He was lost to follow-up and the hardware was never removed. The patient presented to our facility with a periprosthetic subtrochanteric proximal femur fracture just distal to the retained hardware. The DHS was removed and the fracture fixed with an intramedullary nail. The patient healed the fracture and did well.

**Discussion:**

A literature review was performed to highlight the benefits and complications of hardware removal vs. retention. We hope to equip the orthopedic surgeon with the reasons for or against hardware removal to optimize treatment to each patient. In this instance, we recommend hardware removal due to the serious consequences of retained hardware in the adolescent/young adult population.

## 1. Introduction

Orthopedic implants are commonly used in pediatric patients for the treatment of traumatic injuries, developmental hip dysplasia, Legg-Calve-Perthes disease, rotational osteotomies, and slipped capital femoral epiphysis (SCFE) [1]. Current literature indicates that pediatric subtrochanteric fractures are best treated surgically with fixed-angle devices such as a dynamic hip screw, bridge plating, or retrograde elastic nailing [2]. There remains considerable controversy regarding the necessity of the removal of these implants after definitive healing. Literature searches performed through PubMed and ClinicalKey revealed no other case reports detailing a periprosthetic proximal femur fracture about retained hardware in an adolescent patient. The case report argues for hardware removal by highlighting a significant complication secondary to a retained dynamic hip screw in a pediatric patient following a subtrochanteric femur fracture. The authors aim to offer further information on a topic that is still debated due to limited information in the orthopedic literature.

## 2. Case Report

A 20-year-old male was transferred to our tertiary level one trauma center from an outlying community-based hospital following a noncontact basketball injury. Earlier that evening, the patient had been playing basketball at a local church when he jumped for a rebound and experienced severe pain in the left hip and thigh upon landing. He was unable to bear weight. At that time, he was taken to a local emergency department where he underwent immediate evaluation. Following the initial evaluation and review of radiographs, it was determined that the patient had sustained a subtrochanteric periprosthetic femur fracture. At that time, he was transferred to our facility for definitive management.

After further investigation and questioning upon arrival at our facility, the patient reported a 5-6 month history of increasing left thigh pain prior to this injury. He had never received any medical care for the new onset thigh pain and attributed it to overuse. New radiographs obtained at our facility demonstrated a transverse periprosthetic femur fracture at the distal aspect of the previously placed dynamic hip screw. Nine years prior at the age of 11, the patient had been involved in a motor vehicle accident sustaining a subtrochanteric femur fracture which was addressed with reduction and placement of a dynamic hip screw (DHS). The surgery was noted to be performed flawlessly with no complications noted, but little is known about the details of the postoperative course as it was done at an outside facility.

**Figure 1:**
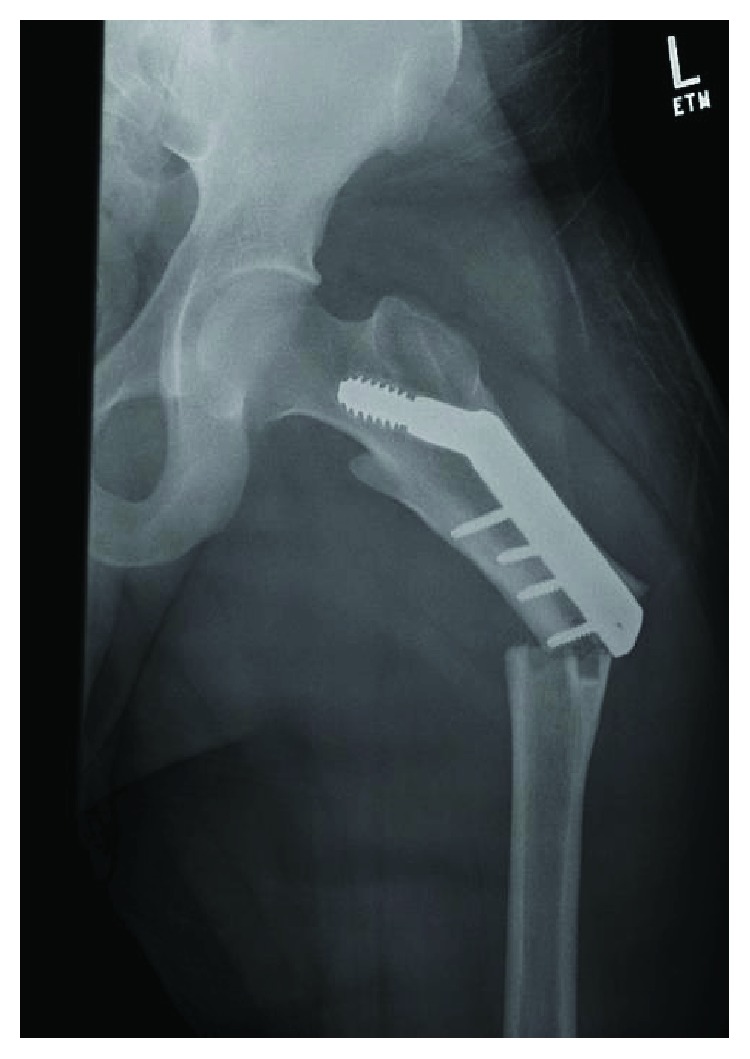
AP view of left hip injury radiographs showing periprosthetic femur fracture with evidence of a potential stress riser at the distal DHS/bone junction, with a significant amount of bony overgrowth of the DHS.

**Figure 2:**
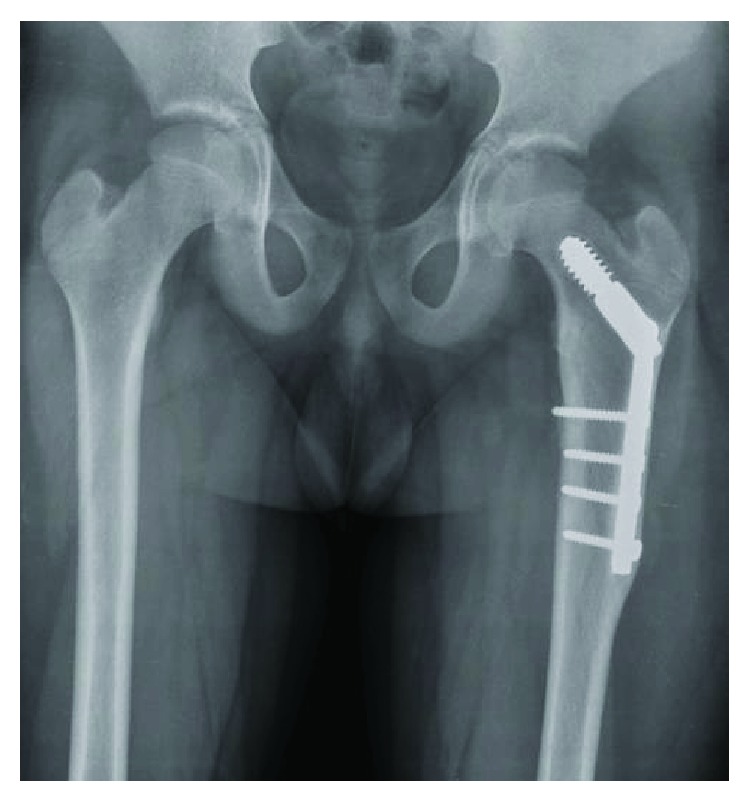
AP view of bilateral proximal femurs taken years prior to injury, showing evidence of stress shielding with significant bony overgrowth of the DHS.

Despite discussions with the index surgeon to eventually remove the hardware, his case was never scheduled and the hardware was retained into skeletal maturity. Close inspection of imaging at the time of repeat injury showed evidence of a potential stress riser at the distal DHS/bone junction, with a significant amount of bony overgrowth of the DHS (Figure 1). Additionally, there was evidence of stress shielding with significant cortical thickening involving the entire lateral cortex seen on radiographs taken prior to injury (Figure 2).

**Figure 3:**
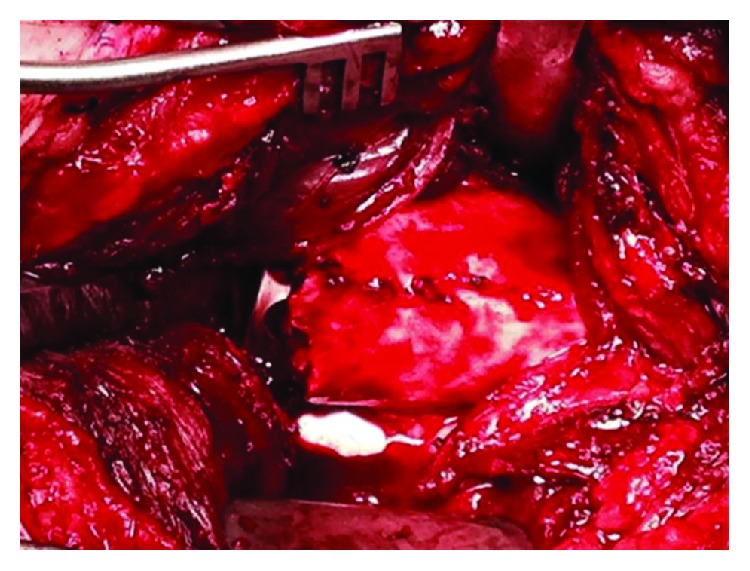
Intraoperative images of the left femur showing extensive bony overgrowth of the implant.

**Figure 4:**
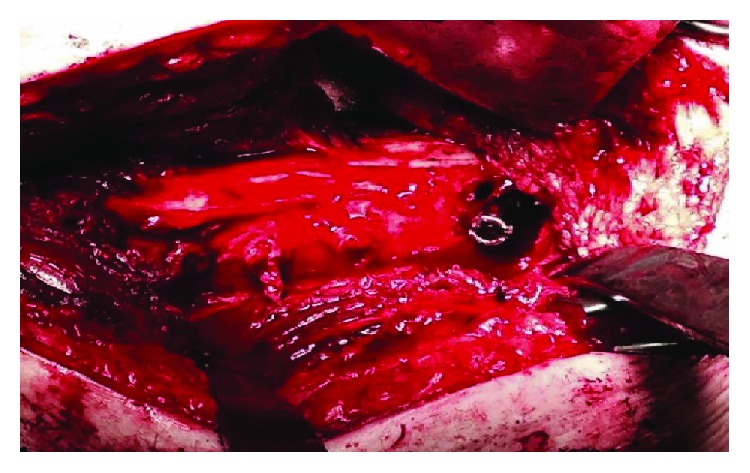
Intraoperative images of the left femur after removal of bony overgrowth and lateral plate with osteotomies. The hip screw is still in place.

**Figure 5:**
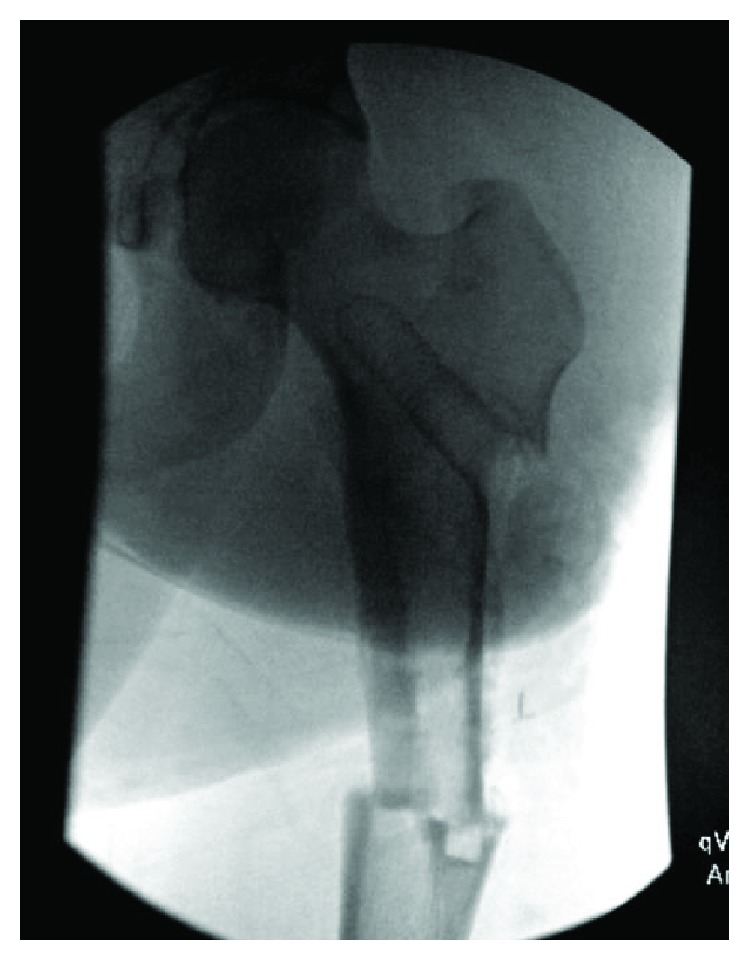
Intraoperative C-arm image of the left femur following implant removal. Notice the thin lateral cortex.

**Figure 6:**
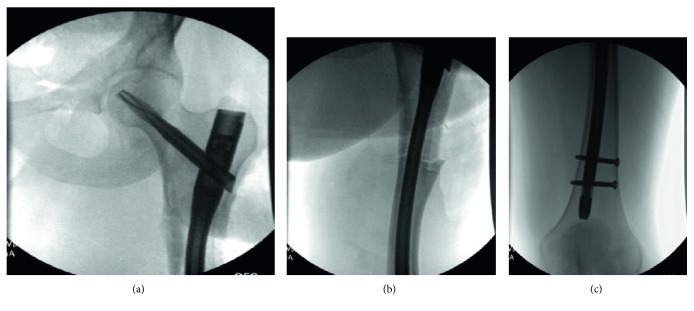
(a-c) Intraoperative C-arm images of the left femur demonstrating a long-reamed cephalomedullary nail with distal interlocking screws.

**Figure 7:**
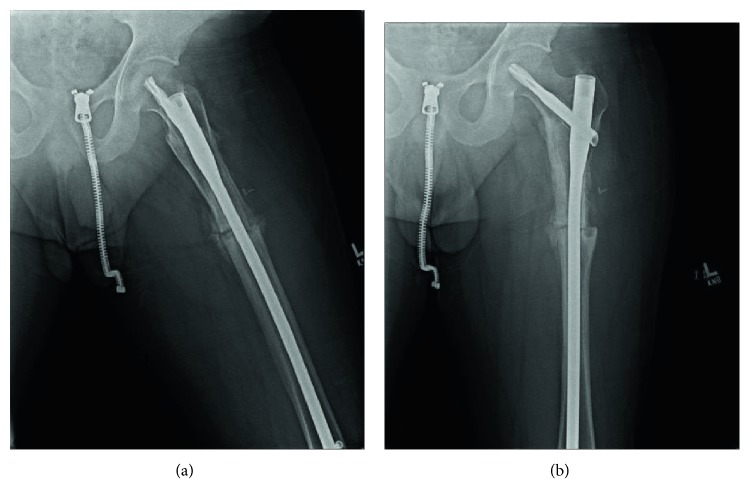
(a and b) Postoperative radiographs showing a healing fracture with abundant callus along the cortex.

The patient was taken to the operating room for explant, fracture reduction, and insertion of a cephalomedullary nail. An extensile approach was necessary to completely expose the retained hardware. Gross intraoperative inspection showed extensive bony overgrowth of the implant (Figure 3). Osteotomies were utilized to remove the bony overgrowth to extract the hardware (Figures 4 and 5). After successful removal of the previous sliding hip screw, the fracture was exposed, reduced, and a long reamed cephalomedullary nail was placed with distal interlocking screws without difficulty (Figures 6(a)–6(c)). Postoperatively, his pain was initially controlled with IV pain medication and he was quickly transitioned to orals only. He was immediately weightbearing as tolerated with the aid of a walker. He progressed quickly with PT, working with them once a day. He was able to walk 120 feet with PT on postoperative day 2 with the use of a walker. He was placed on Lovenox for DVT prophylaxis until he was fully weightbearing using no assistance. He was discharged home on postoperative day #2. He presented to the emergency department two weeks postoperatively complaining of bloody drainage from the incision. The dressing was removed by the ED physician who noted no drainage and was unable to express any blood. The patient was otherwise feeling well. His pain had been controlled, and he denied fevers, chills, nausea, or vomiting. A new dressing was placed and he was discharged home with follow-up with the orthopedic surgeon who performed the case. In outpatient follow-up, the patient continued with Lovenox for DVT prophylaxis as well as physical therapy until he was weightbearing as tolerated without the use of aids. At subsequent postoperative follow-up visits, radiographic imaging revealed complete union of the previous fracture with an abundant amount of bridging callus (Figures 7(a) and 7(b)). The patient reported no other postoperative complications.

## 3. Discussion

A review of the literature demonstrates the controversies of routine implant removal in the pediatric population. Multiple sources support the removal of internal fixation devices. The primary indications for implant removal include pain prevention, implant prominence, infection, stress shielding with late fracture, possibility of malignant degeneration, potential adverse effects on bone growth, late implant infection, metal allergy, risk of corrosion, metal detection, and an increased complication rate at time of future joint reconstruction [1–13].

In a study by Lundeen et al., sixty-eight percent of respondents, who were hip society members and hip reconstruction fellowship directors, recommended that pediatric orthopedic surgeons routinely remove internal fixation from the proximal femur and pelvis [9]. The specific devices that were most concerning to these respondents were blade plates, hip screws and side plates, and intramedullary nails. It was felt that any implants that interfere with the greater trochanter and/or the proximal diaphyseal region of the femur cause subsequent procedures that may be more technically demanding.

Woodcock et al., along with Kanlic and Cruz, felt that delayed implant removal after bone growth over the implant can expose the patient to a secondary procedure of greater magnitude and morbidity and increased risk of fracture at the time of total hip arthroplasty. The former recommended removal of pediatric implants once bone healing has occurred in those at increased risk for future total hip arthroplasty. However, Kanlic and Cruz recommended implant removal in all pediatric femur fracture patients [2].

An additional study by Pate et al. found that bony overgrowth was related to increased operative duration at time of removal. In contrast to popular belief, the amount of bony overgrowth was not related to the timing of implant removal. In this study, the patients who had prolonged surgeries had radiographic evidence of bony overgrowth at just 55 days. This indicates that the timing of implant removal was not as important as radiographic evidence of bony overgrowth. The authors felt that bony overgrowth was more likely to be due to periosteal stripping during the index procedure rather than the timing of implant removal [10].

Rockwood and Wilkins concur with this belief and state in their text that extensile dissection and periosteal stripping during traditional compression plating may lead to bony overgrowth [14].

In contrast to this, research by Kovar et al. found that there was a 28% complication rate in those who had implant removal where the authors deemed the case not medically necessary. In the medically necessary group, which included cases due to infection, mechanical problems, or implant failure, the complication rate was 11.46%. This group had an average time of implant removal of 18 months, whereas the nonmedically necessary group's average time of implant removal was between 2 and 3.5 years. The researchers concluded that the increased time to implant removal played a role in the complexity of implant removal, among other factors [8].

In a meta-analysis by Raney et al., the complication rate for implant removal, excluding SCFE cases, was 6%. The authors of the study could not support or refute routine implant removal in the pediatric population based on their data. There is currently no consensus regarding the need for implant removal in pediatric patients per Kelly et al. [7] They agree with the popular belief that significant bony overgrowth surrounding the entire hardware is likely to require an extensile exposure, potentially making an elective hardware removal more difficult. Greene and Swiontkowski concurred and discouraged routine implant removal except in the pelvis and proximal femur [5].

A web-based study was performed where 273 pediatric orthopedic surgeons and 99 nonpediatric orthopedic surgeons were asked to complete a questionnaire asking them to consider a series of cases and choose whether they would or would not recommend implant removal. Regarding asymptomatic, stainless steel implants in children, 41% recommended removal most or all the time, 36% sometimes, and the remaining 22% recommended removal almost never. The decision to remove an implant was primarily due to its location. Hardware in the proximal femur and around physes was generally removed, while hardware in the pelvis and femoral diaphysis was retained. Another major factor was the age of the patient, with routine removal in younger children and retention in those who were older. More experienced pediatric and general orthopedic surgeons favored routine removal compared with their less experienced colleagues [15].

Davids et al. performed a study consisting of 1223 implants that were implanted in and then removed from 801 children. They were able to extract four risk factors that were predictive of a major complication and one risk factor for minor complication. The four risk factors predictive of a major complication are the following: complication after the initial implant insertion, nonelective indication for implant removal, neuromuscular disease combined with a seizure disorder, and a neuromuscular disorder in a nonambulatory child. Children with all four risk factors had a nearly 15% increased risk of a major complication. Children with a complication following implant insertion were more than three times more likely to have a minor complication following implant removal. This study must be applied to the general pediatric population with caution as 70% of the patients had a neuromuscular disorder [4]. Further recommendations for implant removal were given by Peterson following his own experience combined with a meta-analysis of the current data in 2004. His recommendations state that all Kirschner wires and Steinmann pins, smooth or threaded, should be removed as soon as fracture healing is noted. The potential for migration is too great to leave to chance. Blade plates about the hip should be removed as soon as the fracture is healed and before bony overgrowth occurs. Peterson stated that many of these patients become candidates for reconstructive hip surgery, and the anatomic changes due to the implant make the reconstruction much more difficult. He also recommended that plates on the long bones of the lower extremity be removed as fractures associated with stress shielding of the retained plates can be difficult to treat. Finally, Peterson advises the removal of implants in those that are involved in contact sports as soft tissues adjacent to an implant are more prone to injury and fractures adjacent to an implant may be much more difficult to treat [11].

Another reason cited for implant removal is a deep, late infection. Highland and LaMont reported on six cases of deep, late infection following 63 proximal femoral osteotomies in children with cerebral palsy. The patients presented 7 to 24 months after the index surgery. In another study consisting of 152 pediatric patients, 10 developed deep infections that occurred, on average, 13 months after surgery [13]. Both authors recommended routine removal of the metallic implants in light of these findings. It is of the authors' opinion that it is difficult to recommend implant removal purely based on these findings.

It is the authors' opinion that hardware implanted in the femur of adolescents and young adults should be removed. We believe that the benefits of removal outweigh the risks associated with hardware retention. Prior to hardware removal, a thorough discussion of potential complications should occur. Various articles detail the high complication rate of hardware removal and how these procedures can be much more difficult than expected. Other sources, and our case report, have also documented the long-term complications of retained hardware which are generally more serious complications than hardware removal complications. Some of the major complications of hardware retention include bony overgrowth, stress shielding, stress riser, fracture, hardware failure and migration, and late infection.

This case serves as an example of significant bony overgrowth in a patient greater than 10 years of age who sustained a late periprosthetic femur fracture. The need for implant removal about the proximal femur is not well documented in the reviewed literature.

## 4. Conclusion

The risks associated with both hardware retention and removal will continue to be a point of discussion in the future. Due to the controversy that exists in the orthopedic literature, larger studies are needed over an extended time frame to follow pediatric and young adult patients who have implants removed compared to those who have retained hardware in the proximal femur. It is imperative to educate the patient and family about the known risks and benefits of implant removal vs. retention. The authors' recommend hardware removal for femoral implants in pediatric patients and young adults due to the potential serious complications from retained hardware. This case report highlights the importance of educating the patient and parents about the signs and symptoms of a pending fracture as our patient had prodromal symptoms leading up to the periprosthetic fracture. Prolonged periodic follow-up needs to be recommended for patients with retained hardware. This is no easy task as many young patients are lost to follow-up as they move away from home for school and work.
